# Silent Obstructive Nephrolithiasis Resulting in a Non-functional Kidney

**DOI:** 10.7759/cureus.109687

**Published:** 2026-05-26

**Authors:** Naisargi Patel, Rudolph Bowens

**Affiliations:** 1 Urology, St. Bernards Medical Center, Jonesboro, USA

**Keywords:** asymptomatic kidney stone, bilateral kidney stones, chronic obstruction, cortical thinning, large kidney stone, obstructive hydronephrosis, tubular atrophy

## Abstract

Asymptomatic nephrolithiasis can lead to progressive and irreversible kidney damage. We report a 79-year-old woman with incidentally discovered bilateral kidney stones identified on low-dose computed tomography (CT) imaging performed for lung cancer screening. Imaging revealed a 24 mm left renal stone with severe cortical thinning, suggestive of markedly reduced left renal function, and an 8.7 mm right ureteral stone causing hydronephrosis in a functional kidney. The patient denied flank pain, discomfort, hematuria, or other urinary symptoms. Laboratory evaluation demonstrated preserved renal function with a creatinine of 0.9 mg/dL and an estimated glomerular filtration rate (eGFR) of 65 mL/min/1.73 m². The obstructed right kidney was treated with lithotripsy and ureteral stone removal, while the left kidney was managed conservatively. Guideline-based follow-up and preventive measures were initiated to preserve the remaining renal function. This case highlights that silent obstruction may lead to irreversible renal damage, emphasizing the importance of timely detection and management, particularly in patients with a solitary functioning kidney.

## Introduction

Kidney stone disease is a common condition in the United States. Data from the National Health and Nutrition Examination Survey (NHANES) 2015-2018 survey shows a kidney stone incidence of 2.1% per year affecting approximately 11% of the adult population [1]. While the majority of patients present with flank pain or hematuria, some remain asymptomatic and are discovered incidentally [2]. Silent obstruction may progress unnoticed and can result in hydronephrosis, cortical thinning, and irreversible renal damage. The American Urological Association (AUA) provides recommendations on the management of symptomatic and asymptomatic stones, including criteria for intervention, surveillance, and preventive strategies. Current guidelines support risk-based follow-up and surveillance after stone detection, particularly in patients at increased risk of recurrence or obstruction.

## Case presentation

A 79-year-old woman was referred to the urology clinic after incidental findings on low-dose computed tomography (CT) performed for lung cancer screening. She denied flank pain, hematuria, or urinary symptoms. Laboratory tests, including renal function and urinalysis, were within normal limits as part of her annual physical examination. Serum creatinine was 0.9 mg/dL with an estimated glomerular filtration rate (eGFR) of 65 mL/min/1.73 m².

Imaging

CT revealed an 8.7 mm right ureteral stone with associated hydronephrosis and preserved renal parenchyma (Figure 1).

**Figure 1:**
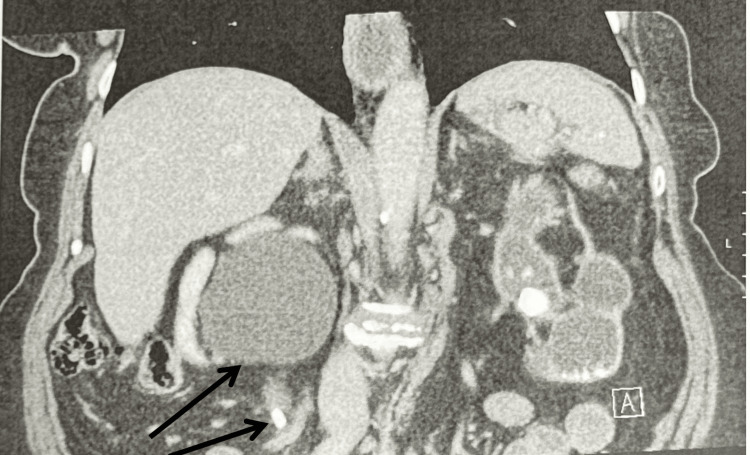
Computed tomography scan showing an 8.7 mm right ureteral stone (arrow) with associated right-sided hydronephrosis (arrow) and preserved renal parenchyma

Additionally, a 24 mm left renal pelvis stone was identified with severe cortical thinning (Figure 2).

**Figure 2:**
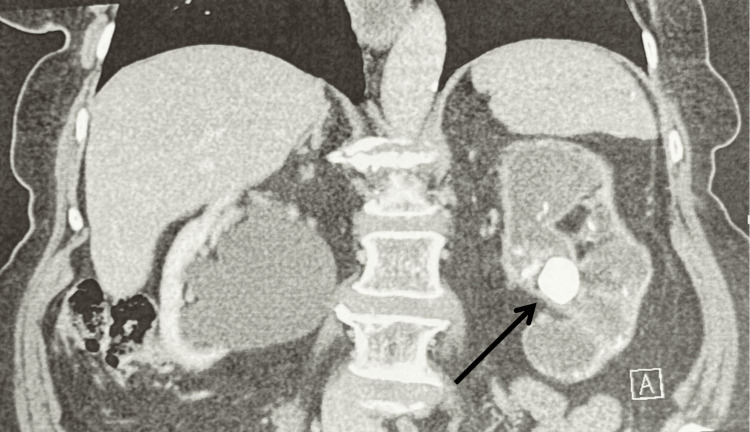
Computed tomography scan showing a 24 mm left renal pelvis stone (arrow) with severe cortical thinning concerning for chronic obstructive renal damage

A kidney, ureter, and bladder (KUB) radiograph confirmed bilateral renal calculi (Figure 3).

**Figure 3:**
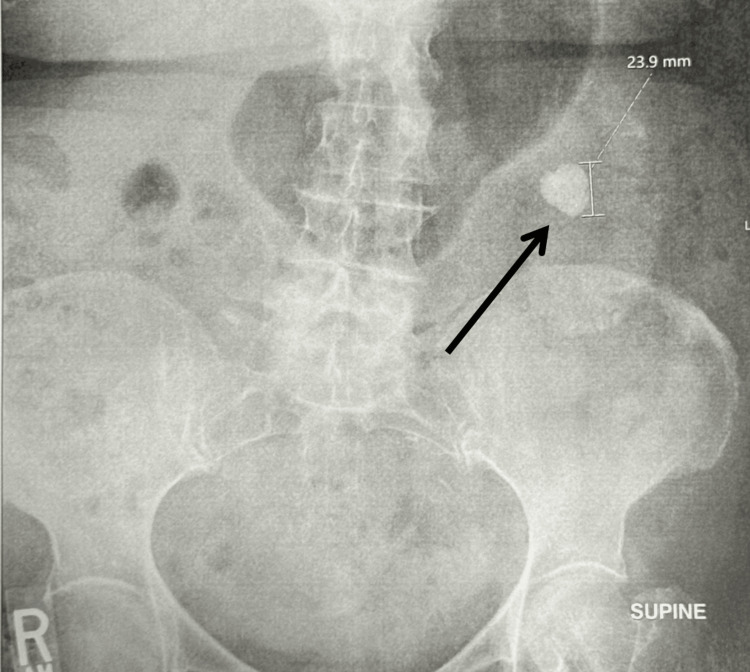
Kidney, ureter, and bladder radiograph confirming bilateral renal calculi, with the arrow indicating the left renal calculus

Overall, these imaging findings were concerning for markedly reduced left renal function, effectively resulting in a solitary functioning right kidney.

The patient underwent right-sided lithotripsy with ureteral stone removal, which resolved hydronephrosis and restored unobstructed urine flow. The left kidney was managed conservatively due to the radiographic evidence of chronic irreversible damage. Post-procedure management included patient education regarding lifestyle modifications, adequate hydration, and dietary measures following AUA Medical Management Guidelines to reduce recurrence risk. Periodic renal ultrasonography was recommended to monitor the right kidney for recurrent obstruction or stone formation as part of risk-based surveillance following stone detection.

## Discussion

Kidney stones affect a substantial proportion of adults in the United States, with a prevalence of approximately 11% and an annual incidence of about 2% [1]. While symptomatic stones often lead to evaluation, asymptomatic stones can remain undetected and, in rare cases, cause chronic obstruction and progressive renal damage.

The natural history of asymptomatic stones is variable [2]. Some remain stable and clinically silent for years, while others gradually enlarge, migrate, or cause asymptomatic obstruction. Chronic obstruction may not produce pain or hematuria, yet it can be sufficient to cause hydronephrosis, cortical thinning, and irreversible renal injury. In this case, the patient's left kidney demonstrated severe cortical thinning associated with a 24 mm stone despite the absence of symptoms or laboratory abnormalities, highlighting the potential severity of undetected nephrolithiasis.

AUA guidelines emphasize that obstructing or symptomatic stones require intervention to relieve obstruction and preserve renal function, particularly in high-risk settings such as a solitary functioning kidney [3]. In contrast, asymptomatic, non-obstructing stones may be managed conservatively with active surveillance and preventive strategies [4]. Following definitive treatment, structured follow-up with imaging surveillance is recommended to identify recurrent stone formation or new obstruction and to guide secondary prevention [4]. Importantly, current guideline recommendations for surveillance and follow-up apply after stone detection, while routine screening imaging is not generally recommended in individuals without a prior history of nephrolithiasis.

Prolonged urinary tract obstruction has been associated with increased intratubular pressure, reduced renal perfusion, tubular atrophy, and interstitial fibrosis, ultimately contributing to irreversible nephron loss [5,6]. These pathophysiologic changes may persist even after relief of obstruction and can occur silently without substantial laboratory abnormalities, as demonstrated in this case.

This case highlights that asymptomatic nephrolithiasis may not be a benign condition and can progress to substantial renal damage if left undetected. Silent obstruction should therefore be recognized as a clinically significant entity, particularly in patients with imaging findings suggestive of a solitary functioning kidney, where delayed diagnosis may contribute to irreversible loss of renal function.

## Conclusions

Asymptomatic nephrolithiasis may result in severe irreversible kidney damage. Chronic obstruction, even in the absence of symptoms, can be associated with cortical thinning and substantial loss of renal function. This case highlights the importance of timely detection, appropriate intervention, and guideline-based follow-up to help preserve renal function, particularly in patients with imaging findings suggestive of a solitary functioning kidney.
